# Health-related quality of life in patients with inoperable malignant bowel obstruction: secondary outcome from a double-blind, parallel, placebo-controlled randomised trial of octreotide

**DOI:** 10.1186/s12885-020-07549-y

**Published:** 2020-10-31

**Authors:** Nikki McCaffrey, Tegan Asser, Belinda Fazekas, Wendy Muircroft, Meera Agar, Katherine Clark, Simon Eckermann, Jessica Lee, Rohit Joshi, Peter Allcroft, Caitlin Sheehan, David C. Currow

**Affiliations:** 1 Deakin Health Economics, Centre for Population Health Research, School of Health and Social Development, Deakin University, Geelong, Victoria Australia; 2 Palliative & Supportive Services, Flinders University, Bedford Park, South Australia Australia; 3 School of Medicine, Flinders University, Bedford Park, South Australia Australia; 4 IMPACCT, Faculty of Health, University of Technology, Ultimo, New South Wales Australia; 5 Australian National Cancer Symptom Trials Group, University of Technology Sydney, Ultimo, New South Wales 2007 Australia; 6 Southern Adelaide Palliative Services, Southern Adelaide Local Health Network, Bedford Park, South Australia Australia; 7 Liverpool Hospital, South West Sydney Local Health District, Liverpool, New South Wales Australia; 8 Cancer & Palliative Care Network, Northern Sydney Local Health District, St Leonards, New South Wales Australia; 9 Northern Clinical School, The University of Sydney, St Leonards, New South Wales Australia; 10 Centre for Health Service Development, Australian Health Services Research Institute, University of Wollongong, Wollongong, New South Wales Australia; 11 Concord Centre for Palliative Care, Concord Repatriation General Hospital, Concord, New South Wales Australia; 12 Medical Oncology, Lyell McEwin Hospital, Adelaide, South Australia Australia; 13 Calvary Heath Care, Kogarah, New South Wales Australia; 14 Wolfson Palliative Care Research Centre, University of Hull, Hull, HU6 7RX England

**Keywords:** Patient-reported outcome measures, Neoplasms, Intestinal obstruction, Terminal care, Palliative care, Randomised controlled trials

## Abstract

**Background:**

This analysis aims to evaluate health-related quality of life (HrQoL) (primary outcome for this analysis), nausea and vomiting, and pain in patients with inoperable malignant bowel obstruction (IMBO) due to cancer or its treatments randomised to standardised therapies plus octreotide or placebo over a maximum of 72 h in a double-blind clinical trial.

**Methods:**

Adults with IMBO and vomiting recruited through 12 services spanning inpatient, consultative and community settings in Australia were randomised to subcutaneous octreotide infusion or saline. HrQoL was measured at baseline and treatment cessation (EORTC QLQ-C15-PAL). Mean within-group paired differences between baseline and post-treatment scores were analysed using Wilcoxon Signed Rank test and between group differences estimated using linear mixed models, adjusted for baseline score, sex, age, time, and study arm.

**Results:**

One hundred six of the 112 randomised participants were included in the analysis (*n* = 52 octreotide, *n* = 54 placebo); 6 participants were excluded due to major protocol violations. Mean baseline HrQoL scores were low (octreotide 22.1, 95% CI 14.3, 29.9; placebo 31.5, 95% CI 22.3, 40.7). There was no statistically significant within-group improvement in the mean HrQoL scores in the octreotide (*p* = 0.21) or placebo groups (*p* = 0.78), although both groups reported reductions in mean nausea and vomiting (octreotide *p* < 0.01; placebo *p* = 0.02) and pain scores (octreotide *p* < 0.01; placebo *p* = 0.03). Although no statistically significant difference in changes in HrQoL scores between octreotide and placebo were seen, an adequately powered study is required to fully assess any differences in HrQoL scores.

**Conclusion:**

The HrQoL of patients with IMBO and vomiting is poor. Further research to formally evaluate the effects of standard therapies for IMBO is therefore warranted.

**Trial registration:**

Australian New Zealand Clinical Trials Registry ACTRN12608000211369 (date registered 18/04/2008)

**Supplementary Information:**

The online version contains supplementary material available at 10.1186/s12885-020-07549-y.

## Background

Bowel obstruction due to cancer or its treatments (malignant bowel obstruction (MBO)) is prevalent in the advanced stages of illness [1, 2]. For many, this is an inoperable problem due to multi-level obstruction, likely intolerance of the catabolic insult of surgery due to poor health, peritoneal carcinomatosis, complications from previous intra-abdominal surgeries or a combination of these contraindications. In this setting, good symptom control, particularly for nausea and vomiting, becomes the overarching therapeutic goal [3]. For most people with inoperable malignant bowel obstruction (IMBO), prognosis is very poor [3]. Interventions that predictably and quickly reduce the frequency and volume of vomiting are required. However, to date, there are no registered medications for the symptomatic treatment of IMBO [4].

An adequately powered randomized, double-blind, placebo-controlled trial evaluating the net effect of adding octreotide (a somatostatin analogue) or placebo to standardised therapies (ranitidine, dexamethasone, low dose subcutaneous hydration) in people with IMBO [4] failed to demonstrate a statistically significant or clinically important difference in the number of patient-reported days free of vomiting at 72 h. Joint consideration of the primary, secondary and safety outcomes, and patient net clinical benefit is recommended when interpreting the findings of such well-designed clinical trials [5]. Although there were no statistically significant differences between groups for almost all of the secondary outcomes, participants receiving octreotide experienced a reduction in the number of episodes of vomiting compared with placebo (incidence rate ratio 0.40, 95% CI 0.19–0.86, *p*-value = 0.02). At the same time, people in the octreotide arm were twice as likely to be administered hyoscine butylbromide each day compared with placebo (*p*-value < 0.01), possibly due to more colicky pain, greater pain severity or both [4]. Given these differences, octreotide may have distinctive effects on health-related quality of life (HrQoL) domains and overall quality of life compared with placebo.

This paper reports the first analysis of HrQoL in a randomized trial comparing octreotide with placebo. To date, the previously published studies examining this issue [6] in similar populations have been mixed [7–9]. Matulonis and colleagues [8] reported no statistically significant differences among any HrQoL scores (measured using the EORTC QLQ-C30/ OV28) in people with advanced cancer and inoperable, clinically or radiographically diagnosed bowel obstruction treated with octreotide up to 12 months duration. In contrast, Shima et al. [9] found that some aspects of HrQoL (measured using a subjective self-administered questionnaire), such as alleviation of nausea and vomiting and enjoyment of recreational activities, did improve after 6 days of octreotide therapy in patients with malignant bowel obstruction refractory to other medical treatment. Finally, Hisanaga and colleagues [7] reported a statistically significant improvement in overall HrQoL (measured using a face scale, *p* < 0.001) in palliative patients with IMBO treated with octreotide for 3 days but, of note, no improvement in pain. However, as all of these results were derived from lower levels of evidence more prone to bias, predominantly due to a lack of control group, the findings should be interpreted cautiously [10]. Recently, Obita et al. [11] conducted a systematic review assessing the efficacy of somatostatin analogues for symptomatic treatment of nausea and vomiting in people with advanced cancer and malignant bowel obstruction and concluded whilst there was low level evidence of benefit, higher quality studies with a low risk of bias did not demonstrate benefit, consistent with the results from this study.

More generally, there is very little published data on the HrQoL of people with IMBO using validated instruments [12–14]. Observational data (*n* = 35) reported by Selby and colleagues indicate patients presenting with malignant bowel obstruction from metastatic intra-abdominal disease have very poor HrQoL and a high symptom burden, as measured with the Rotterdam Symptom Checklist (RSCL) and Edmonton Symptom Assessment Scale (ESAS) respectively [12]. Detailed analysis of the HrQoL data collected during the octreotide study conducted by Currow and colleagues [4] using the European Organisation for Research and Treatment of Cancer Quality of Life Questionnaire (EORTC) QLQ-C15-PAL helps address this knowledge gap.

The primary aim of this current analysis of prospectively collected data from the octreotide randomised controlled trial (RCT) was to evaluate whether there is a difference in overall HrQoL between participants treated with octreotide and placebo. The secondary aims were to compare the pain and nausea and vomiting dimensions of HrQoL in the treatment arms and evaluate the HrQoL of people with IMBO. A preliminary analysis of these data has been previously presented at the 15th World Congress of the European Association for Palliative Care, 18–20 May 2017, Madrid, Spain [15].

## Methods

### Setting

The Palliative Care Clinical Studies Collaborative (PaCCSC) is a national research network established in 2006 with funding from the Australian Government, conducting robust, multi-site, RCTs investigating the effectiveness and cost-effectiveness of symptom treatments in palliative care [16]. This PaCCSC RCT was conducted between August 2008 and May 2012 and was approved by the ethics committees of 12 participating sites spanning all clinical settings (inpatient, consultative, community) across Australia. Participants provided written informed consent. The study was registered in the Australian New Zealand Clinical Trials Registry (ACTRN12608000211369, <https://www.anzctr.org.au/Trial/Registration/TrialReview.aspx?id=82724>). Full details of the trial are reported elsewhere [4] and briefly summarised below.

### Trial design

Adults with IMBO (diagnosed on clinical grounds by two independent medical practitioners) and vomiting for whom further anti-cancer therapies were not immediately appropriate were randomised to a subcutaneous infusion of octreotide (600 mg/24 h) or normal saline (placebo) for a maximum of 72 h. Trained palliative care research nurses obtained consent and enrolled potential participants identified by clinicians (emergency, surgical, general medicine, and oncology departments and palliative care services) in participating institutions and their associated community teams. The eligibility criteria are summarized in Table 1.

**Table 1:** Eligibility criteria [4]

Inclusion criteria	Exclusion criteria
Age > 18 years	Previous adverse reaction to any of the study medications
Advanced cancer	Australia-modified Karnofsky Performance Score < 30 at study entry
Disease-modifying therapy (surgery, chemotherapy, radiotherapy, hormone therapy, biological/targeted therapies) is unlikely to change the bowel obstruction.	Participated in a clinical study of a new chemical entity < 1 month before study entry
Presents with clinically confirmed bowel obstruction with vomiting that precipitates change in clinical care or a hospital admission.	Calculated creatinine clearance < 10 mL/minute
A partial or complete bowel obstruction for which immediate surgery is not indicated (confirmed by two consultant-level medical practitioners).	Clinically significant cirrhosis (documented)
Able to complete assessments and comply with the study procedures	Feeding or venting gastrostomy or jejunostomy
Able to give fully informed written consent	
Not currently on octreotide	

Randomised treatment was combined with standardised therapies which included regular parenteral dexamethasone (8 mg/day), ranitidine (200 mg/24 h), and hydration (10–20 mL/kg/day unless overtly dehydrated at study entry) [4]. Standardised “as-needed” therapies were available for managing expected symptoms during the study period including; hyoscine butylbromide for colicky abdominal pain, haloperidol for nausea, and parenteral opioids for pain. Participants were randomized in blocks of four by site in a 1:1 ratio and randomization schedules were developed for each site using random number tables, generated centrally [4]. Site pharmacists, uninvolved in the participants’ care, opened the treatment schedules. Syringes were identical in colour and volume. Participants, assessors and clinical staff were blinded to treatment allocations. The primary outcome was the number of patient-reported days free of vomiting at 72 h. Secondary outcomes, also measured at 72 h, included the number of patient-reported episodes of vomiting, episodes of vomiting per day, survival, level of nausea (numerical rating scale), average pain score (Brief Pain Inventory [BPI]) [17], functional status (Australia-modified Karnofsky Performance Status [AKPS]) [18], protocol-defined as-needed symptom control medications, patient-rated Global Impression of Change (GIC) and HrQoL [4, 19].

### Health-related quality of life (HrQoL)

Health-related quality of life was measured using the EORTC QLQ-C15-PAL questionnaire which was administered at baseline and treatment cessation. The EORTC QLQ-C15-PAL is a shortened version of the widely used cancer-specific HrQoL measure, the EORTC QLQ-C30 [20], and was specifically developed for palliative care. The EORTC QLQ-C15-PAL questionnaire consists of 14 items, each with four possible responses (not at all = 1, a little = 2, quite a bit = 3, and very much = 4), and an overall HrQoL rating scale with seven categories ranging from 0 (very poor) to 7 (excellent) [21]. The 14 items are grouped into two functional scales (physical and emotional), five single-item symptom scales (dyspnoea, insomnia, appetite loss, constipation, nausea and vomiting) and two multi-item symptom scales (pain and fatigue). A scoring algorithm is used to convert the response categories to a score (0–100) [22, 23]. Lower scores on the functional scales indicate reduced levels of functioning, whereas on the symptom scales, lower scores indicate a reduced symptom burden. Note the EORTC-QLQ-C15 questionnaire does not produce an overall total score derived from all the items.

### Analysis

Analysis of the HrQoL data was undertaken on an intention-to-treat basis. Missing value patterns were analyzed using missing value analysis in SPSS® (SPSS, Inc., Chicago, IL) to evaluate whether there were systematic differences between available and missing values. Following Consolidated Standards of Reporting Trials (CONSORT) statement recommendations [24, 25], multiple imputation was used to estimate participant-level missing HrQoL data with a fully conditional specification model. All variables in the dataset were included in the imputation model and 20 imputations were performed [26, 27]. Statistical analyses were conducted using Statistical Package for Social Sciences for Windows versions 23.0 and 24.0 (SPSS, Inc., Chicago, IL).

The pre-specified, null hypothesis for the primary outcome in the HrQoL analysis was that there would be no difference in overall HrQoL between treatment arms. A difference of 10 points or more on the 100 point overall HrQoL scale (“How would you rate your quality of life during the past week”) was considered clinically meaningful based on earlier studies [28–31]. The alternative hypotheses for the secondary outcomes, were that there would be a difference in the nausea and vomiting and pain dimensions between treatment arms, favouring octreotide. A difference of 13 points or more on the 100 point scale was considered clinically meaningful for the EORTC QLQ-C15-PAL pain and nausea and vomiting dimensions based on a recent study reporting minimal clinically important differences (MCID) estimated in patients with bone metastases undergoing radiotherapy [30].

Descriptive statistics were calculated for clinico-demographic variables and HrQoL scores. As the baseline HrQoL scores were non-normally distributed (Kolmogorov-Smirnov test, *p* < 0.05), differences between the two groups at baseline were assessed using the non-parametric Mann Whitney U test [32]. Within-group differences between baseline and post-treatment HrQoL scores were analysed using the Wilcoxon Signed Rank test [32]. Estimated marginal means (maximum likelihood) and the adjusted mean difference between overall HrQoL scores (primary outcome) and pain and nausea and vomiting scores (secondary outcomes), with associated 95% CI and *p*-values, were calculated using a linear mixed effects model (LMM) with a random slope, treatment group and time as fixed effects, and age, gender and baseline scores as covariates [33]. A series of exploratory LMMs were conducted to confirm the fixed and random effects and optimal covariance structure. Akaike’s Information Criterion (AIC) was used to aid identification of the best-fitting model [34]. The significance level in secondary analyses was set at *p* < 0.05 with two-sided significance tests.

## Results

**Fig. 1:**
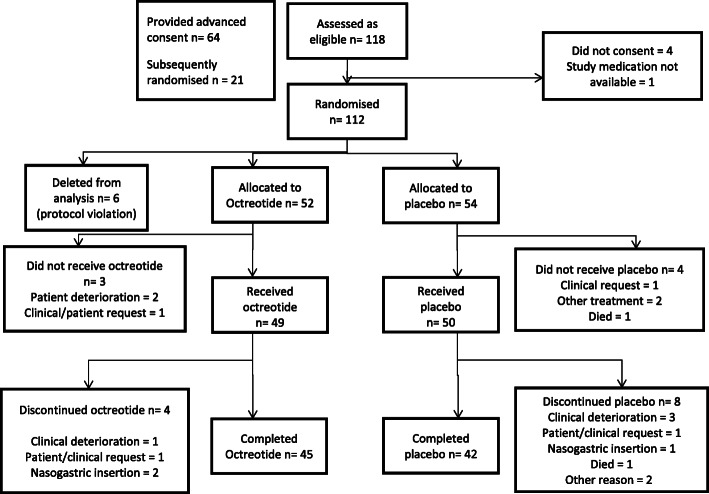
CONSORT participant flow diagram [4]. Reprinted from *Journal of Pain and Symptom Management*, Volume 49, Issue 5, David C. Currow, Stephen Quinn, Meera Agar et al., Double-Blind, Placebo-Controlled, Randomized Trial of Octreotide in Malignant Bowel Obstruction, Pages 814–821, Copyright (2015), with permission from Elsevier. 10.1016/j.jpainsymman.2014.09.013

Overall, 112 participants were randomised, although six participants were excluded from the analysis due to major protocol violations of concomitant treatments (Fig. 1). The intended cohort was recruited as planned. Of the 106 participants included in the analysis, 52 were randomised to octreotide and 54 to placebo. There were no deaths in either group during the study.

In the octreotide group, the mean age was 62.9 (SD 13.6), and 90.4% of participants were female, whilst in the placebo group, the mean age was slightly higher (66.3 (SD 12.2)), and 70.4% of participants were female (Table 2). For both groups, multi-level small bowel obstruction was the most common bowel obstruction, affecting 34 participants (65.4%) in the octreotide group and 34 (63.0%) in the placebo group. The median AKPS score in both groups was 50 (requiring considerable assistance). At baseline, overall HrQoL scores were low (octreotide 22.1, 95% CI 14.3, 29.9; placebo 31.5, 95% CI 22.3, 40.7) whilst not statistically higher in the placebo arm (*p*-value = 0.12). Given complete case and multiple imputation descriptive analyses were similar only complete case results are presented below. The multiple imputation descriptive analyses are provided in Table A1, Additional file 1.

**Table 2:** Baseline participant demographics and clinical characteristics

Characteristic	Octreotide ***N*** = 52	Placebo ***N*** = 54
Age in years, mean (SD)	62.9 (13.6)	66.3 (12.2)
Gender, female, n (%)	47 (90.4)	38 (70.4)
Performance status (AKPS), median (IQR)	50 (40–60)	50 (40–60)
BPI pain score, median (IQR)	3 (1–5)	4 (1.25–5)
Nausea^a^, median (IQR)	2 (1–2)	1(1–2)
Level of bowel obstruction		
Gastric outlet/duodenal, n (%)	5 (9.6)	9 (16.7)
Small bowel/ multi-level, n (%)	34 (65.4)	34 (63.0)
Large bowel, n (%)	2 (3.9)	3 (5.6)
Indeterminate, n (%)	11 (21.2)	8 (14.8)
EORTC QLQ-C15-Pal overall quality of life score, mean (SD)	22.1 (24.3)	31.5 (27.2)

*AKPS* Australia-modified Karnofsky Performance Status scale, scores range from 0 to 100 where a higher score indicates better performance in terms of work, activity and self-care, *BPI* Brief Pain Inventory, scores range from 0 to 10 where higher score represents more pain, *EORTC QLQ-C15-Pal* The European Organization for Research and Treatment of Cancer quality of life palliative care questionnaire, scores range from 0 to 100 and higher scores represent better QOL, *IQR* Inter-quartile range, *SD* Standard deviation

^a^nausea numerical rating scale ranges from 0 to 5, where a higher score indicates more nausea

Overall, 81 (76.4%) participants attempted the baseline EORTC QLQ-C15-PAL questionnaire and 67 (63.2%) participants attempted the questionnaire at treatment cessation. Only 42 participants (39.6%) had no missing responses for either questionnaire. Response rates for the EORTC QLQ-C15-PAL functional, symptom and overall HrQoL rating scales are summarised in Table A2, Additional file 2. Total response rates for the individual questionnaire items varied between 67.0% (*n* = 71) and 76.4% (*n* = 81) at baseline and 51.9% (*n* = 55) and 63.2% (*n* = 67) post-treatment, and were similar between study arms. In both groups, there was a higher response rate at baseline compared to treatment cessation.

### Health-related quality of life scores

Baseline and post-treatment EORTC QLQ-C15-PAL scores are presented in Table 3.

**Table 3:** Baseline and post-treatment EORTC QLQ-C15-PAL scores

EORTC QLQ-C15-PAL scale^**a**^	Octreotide (***N*** = 52) mean scores (95% CI)			Placebo (***N*** = 54) mean scores (95% CI)		
	Baseline	Post-treatment	***p***-value	Baseline	Post-treatment	***p***-value
Overall quality of life^b^	22.08 (14.32, 29.85) *n* = 40	30.81 (22.57, 39.05) *n* = 33	0.205	31.48 (22.26, 40.70) *n* = 36	32.22 (21.27, 43.18) *n* = 30	0.778
Physical functioning^b^	27.69 (19.69, 35.69) *n* = 39	32.44 (22.51, 42.36) *n* = 30	0.204	31.17 (22.80, 39.53) *n* = 40	30.00 (19.70, 40.27) *n* = 30	0.831
Emotional functioning^b^	57.65 (47.35, 67.94) *n* = 36	71.80 (60.78, 82.82) *n* = 26	0.059	55.25 (45.26, 65.24) *n* = 35	63.22 (50.20, 76.25) *n* = 29	0.074
Fatigue^c^	74.09 (67.21, 80.97) *n* = 39	71.88 (63.37, 80.40) *n* = 32	0.107	74.10 (67.84, 80.35) *n* = 39	71.88 (60.27, 83.48) *n* = 32	0.965
Nausea & vomiting^c^	**73.18 (62.34, 84.01)** ***n*** **= 41**	**40.70 (27.56, 53.83)** ***n*** **= 34**	**0.001**	**60.53 (50.55, 70.51)** ***n*** **= 38**	**35.95 (23.63, 48.26)** ***n*** **= 32**	**0.016**
Pain^c^	**60.16 (49.33, 71.00)** ***n*** **= 41**	**43.94 (32.23, 55.65)** ***n*** **= 33**	**0.007**	**55.42 (45.01, 65.83)** ***n*** **= 40**	**42.93 (30.48, 55.38)** ***n*** **= 33**	**0.026**
Dyspnoea^c^	40.65 (29.74, 51.56) *n* = 41	32.32 (21.88, 42.76) *n* = 33	0.077	**36.75 (27.89, 45.62)** ***n*** **= 39**	**27.08 (14.34, 39.83)** ***n*** **= 32**	**0.008**
Insomnia^c^	**45.83 (34.59, 57.08)** ***n*** **= 40**	**33.33 (22.28, 44.39)** ***n*** **= 33**	**0.013**	46.67 (34.89, 58.44) *n* = 40	47.92 (32.67, 63.16) *n* = 32	0.868
Appetite loss^c^	**74.17 (62.98, 85.36)** ***n*** **= 40**	**56.25 (43.50, 69.00)** ***n*** **= 32**	**0.034**	**82.05 (74.27, 89.83)** ***n*** **= 39**	**57.58 (43.63, 71.52)** ***n*** **= 33**	**0.002**
Constipation^c^	**67.50 (56.05, 78.95)** ***n*** **= 40**	**50.00 (36.19, 63.81)** ***n*** **= 34**	**0.027**	**67.50 (56.31, 78.69)** ***n*** **= 40**	**52.53 (38.35, 66.71)** ***n*** **= 33**	**0.027**

Bolded numbers indicate statistically significant differences

*HrQoL* Health-related quality of life

^a^mean unadjusted within-group paired differences between baseline and post-treatment scores were analysed using the Wilcoxon Signed Rank test. ^b^ higher scores represent better outcomes; ^c^ higher scores represent greater symptom burden

There was no statistically significant within-group improvement in the mean HrQoL scores in the octreotide (*p* = 0.21) or placebo groups (*p* = 0.78) (Table 3). Both groups reported statistically significant and clinically relevant reductions in mean nausea and vomiting (octreotide *p* < 0.01; placebo *p* = 0.02) and pain scores (octreotide *p* < 0.01; placebo *p* = 0.03). Post hoc analysis of within-group changes in the remaining EORTC QLQ-C15-PAL scores suggested scores generally improved over the duration of the study: appetite loss and constipation for both groups, sleep in the octreotide group and dyspnoea in the placebo group.

Differences between groups were analysed using linear mixed models, adjusting for baseline HrQoL scores, sex and age (evidence in the literature suggests older respondents and females have poorer health generally [35], time and study arm. In the exploratory analyses, group by time interaction was not statistically significant (most post-treatment scores were collected on Day 3) and so was removed from the final model. Table 4 summaries the adjusted, estimated marginal mean post-treatment scores and between-group differences.

**Table 4:** Adjusted estimated marginal mean EORTC QLQ-C15-PAL post-treatment scores

Scales Mean (95% CI)	Octreotide (***N*** = 52)	Placebo (***N*** = 54)	Mean difference (95% CI)	***P***-value
Overall HrQoL^a^	35.31 (21.68, 48.94)	34.86 (21.30, 48.41)	0.45 (−10.35, 11.26)	0.935
Physical functioning^a^	33.97 (21.30, 46.64)	31.01 (20.31, 41.69)	2.97 (−6.52, 12.46)	0.539
Emotional functioning^a^	60.30 (45.18, 75.42)	58.95 (46.22, 71.67)	1.36 (−10.40, 13.12)	0.821
Fatigue^b^	73.58 (60.28, 86.87)	72.88 (59.75, 86.00)	0.70 (−10.07, 11.46)	0.899
Nausea & vomiting^b^	51.45 (33.98, 68.91)	47.79 (32.39, 63.18)	3.66 (−10.35, 17.68)	0.608
Pain^b^	56.38 (40.27, 72.49)	55.53 (42.61, 68.44)	0.85 (− 11.08, 12.79)	0.888
Dyspnoea^b^	31.67 (17.77, 45.54)	29.76 (17.00, 42.53)	1.89 (−9.69, 13.48)	0.748
Insomnia^b^	41.00 (26.17, 55.84)	48.14 (34.14, 62.14)	− 7.14 (− 20.88, 6.61)	0.308
Appetite loss^b^	52.10 (34.34, 69.87)	52.09 (34.90, 69.28)	0.16 (−14.23, 14.26)	0.998
Constipation^b^	49.45 (32.91, 65.99)	51.47 (37.22, 65.72)	−2.02 (−16.22, 12.19)	0.780

*HrQoL* Health related quality of life

^a^higher scores represent better outcomes

^b^higher scores represent greater symptom burden

The mean adjusted difference between the two treatment arms for HrQOL was neither statistically significant nor clinically important (0.5, 95% CI -10.4, 11.3). Similar between group results were observed for the nausea and vomiting (3.7, 95% CI -10.4, 17.7) and pain scores (0.9, 95% CI -11.1, 12.8).

## Discussion

The findings from this pre-specified analysis of prospectively collected HrQoL data from the PaCCSC octreotide RCT suggest the overall quality of life of patients with IMBO and vomiting is very poor. Health-related quality of life, physical and emotional functioning and symptoms measured using the EORTC QLQ-C15-PAL improved over the study period for all participants. Participants reported experiencing less nausea and vomiting, pain, appetite loss and constipation post-treatment, irrespective of treatment allocation. This may reflect the quality of care delivered during the study, the natural history of IMBO and the efficacy of the standardised therapies which included ranitidine and dexamethasone. These therapies provide promising opportunity for further exploration.

Although most symptom scale scores improved, there was no statistically significant improvement in the overall quality of life suggesting other aspects outside of those captured by the EORTC QLQ-C15-PAL influence quality of life [36]. Overall, fatigue, appetite loss and nausea and vomiting were the worst symptoms at baseline, similar to the most predominant symptoms on the ESAS scale in a prospective study of 35 patients admitted to hospital with malignant bowel obstruction (appetite loss, fatigue, drowsiness) [12].

Adding subcutaneous octreotide to standardized therapies did not result in meaningful improvements in HrQoL, pain or nausea and vomiting compared with placebo in this setting and timeframe. The HrQoL endpoints were secondary outcomes and the study likely lacked adequate statistical power to assess any differences in the impact of octreotide and placebo on HrQoL. An estimated sample size of 248 participants would be needed to detect a clinically meaningful between-group difference of 10 points in the overall HrQoL domain, with a power of 90% and an alpha level of 0.05.

Strengths of this study include standardization of the supportive care delivered in both arms using the best available evidence [4]. The randomized, double-blind, placebo-controlled trial, adequately powered for the primary outcome, was conducted across a range of clinical practices reflecting the target population seen in hospice, palliative care, and oncology settings, augmenting external validity. The EORTC QLQ-C15-PAL is a brief, yet comprehensive, validated instrument measuring HrQoL, developed specifically for the palliative care setting [37]. The HrQoL data are derived from RCT evidence, minimizing potential sources of bias associated with lower levels of evidence [6].

Similar to other studies involving a palliative care population [21], there was a sizable proportion of missing values in the data, although patterns of missing values between the study groups were similar. Systematic differences between available and missing baseline values could not be excluded as participants with a higher performance status (as measured by the AKPS) appeared more likely to complete baseline questionnaires, indicating that physically unwell patients were less likely to respond. Post-treatment questionnaire response rates were lower than baseline. Typically, people with a life-limiting illness receiving palliative care have poor health status which declines over time and fatigue may be problematic in this frail population, leading to a higher non-response rate compared with other study populations. Despite these caveats, multiple imputation, a robust statistical approach for dealing with missing data [26], was used to minimise any potential bias due to missing values.

## Conclusions

The HrQoL of patients with IMBO and vomiting is generally poor. Despite this, the findings from this study suggest HrQoL can be improved over a relatively short period of time with appropriate care. Formal evaluation of the effects of ranitidine, dexamethasone, both or neither in this setting is warranted given the improvements in nausea and vomiting, pain and HrQoL domains in the study participants.

## Supplementary Information

**Additional file 1: Table A1.** Baseline and post-treatment EORTC QLQ-C15-PAL mean scores (multiple imputation). Baseline and post-treatment EORTC QLQ-C15-PAL mean scores for completed items and multiple imputation.

**Additional file 2: Table A2.** Baseline and post-treatment EORTC QLQ-C15-PAL completion rates.

## Data Availability

Professor David C Currow has full control of all primary data. These data are available for review on request to Professor Currow (email David.Currow@uts.edu.au; phone + 61 2 95145967).

## Abbreviations

AIC: Akaike’s Information Criterion
AKPS: Australia-modified Karnofsky Performance Status
CI: Confidence intervals
CONSORT: Consolidated Standards of Reporting Trials
EORTC QLQ-C15-PAL: European Organisation for Research and Treatment of Cancer Quality of Life Questionnaire C15 Palliative
ESAS: Edmonton Symptom Assessment Scale
GIC: Global Impression of Change
HrQOL: Health-related quality of life
IMBO: Inoperable malignant bowel obstruction
IQR: Interquartile range
LMM: Linear mixed effects model
MBO: Malignant bowel obstruction
MCID: Minimal clinically important difference
PaCCSC: Palliative Care Clinical Studies Collaborative
RCT: Randomised controlled trial
RSCL: Rotterdam Symptom Checklist
SD: Standard deviation

## References

[1] Tuca A, Guell E, Martinez-Losada E, Codorniu N (2012). Malignant bowel obstruction in advanced cancer patients: epidemiology, management, and factors influencing spontaneous resolution. Cancer Manag Res.

[2] Mercadante S, Casuccio A, Mangione S (2007). Medical treatment for inoperable malignant bowel obstruction: a qualitative systematic review. J Pain Symptom Manag.

[3] Ripamonti C, Twycross R, Baines M, Bozzetti F, Capri S, De Conno F, Gemlo B, Hunt TM, Krebs HB, Mercadante S, Schaerer R, Wilkinson P (2001). Clinical-practice recommendations for the management of bowel obstruction in patients with end-stage cancer. Support Care Cancer.

[4] Currow DC, Quinn S, Agar M, Fazekas B, Hardy J, McCaffrey N, Eckermann S, Abernethy AP, Clark K (2015). Double-blind, placebo-controlled, randomized trial of octreotide in malignant bowel obstruction. J Pain Symptom Manag.

[5] Pocock SJ, Stone GW (2016). The primary outcome fails — what next?. N Engl J Med.

[6] Kindl K, Good P (2015). Evidence-based palliative care 13 years on: has anything changed?. J Palliat Care.

[7] Hisanaga T, Shinjo T, Morita T, Nakajima N, Ikenaga M, Tanimizu M, Kizawa Y, Maeno T, Shima Y, Hyodo I (2010). Multicenter prospective study on efficacy and safety of octreotide for inoperable malignant bowel obstruction. Jpn J Clin Oncol.

[8] Matulonis UA, Seiden MV, Roche M, Krasner C, Fuller AF, Atkinson T, Kornblith A, Penson R (2005). Long-acting octreotide for the treatment and symptomatic relief of bowel obstruction in advanced ovarian cancer. J Pain Symptom Manag.

[9] Shima Y, Ohtsu A, Shirao K, Sasaki Y (2008). Clinical efficacy and safety of octreotide (SMS201-995) in terminally ill Japanese cancer patients with malignant bowel obstruction. Jpn J Clin Oncol.

[10] Wang JJ, Attia J (2010). Study designs in epidemiology and levels of evidence. Am J Ophthalmol.

[11] Obita GP, Boland EG, Currow DC, Johnson MJ, Boland JW (2016). Somatostatin analogues compared with placebo and other pharmacologic agents in the management of symptoms of inoperable malignant bowel obstruction: a systematic review. J Pain Symptom Manage.

[12] Selby D, Wright F, Stilos K, Daines P, Moravan V, Gill A, Chakraborty A (2010). Room for improvement? A quality-of-life assessment in patients with malignant bowel obstruction. Palliat Med.

[13] Naghibi M, Smith TR, Elia M (2015). A systematic review with meta-analysis of survival, quality of life and cost-effectiveness of home parenteral nutrition in patients with inoperable malignant bowel obstruction. Clin Nutr.

[14] Lee YC, Jivraj N, O'Brien C, Chawla T, Shlomovitz E, Buchanan S, Lau J, Croke J, Allard JP, Dhar P, Laframboise S, Ferguson SE, Dhani N, Butler M, Ng P, Stuart-McEwan T, Savage P, Tinker L, Oza AM, Lheureux S (2018). Malignant bowel obstruction in advanced gynecologic cancers: an updated review from a multidisciplinary perspective. Obstet Gynecol Int.

[15] McCaffrey N, Asser T, Fazekas B, Muircroft W, Clark K, Currow D (2017). Health-related quality of life in patients with inoperable malignant bowel obstruction: secondary endpoint from the double-blind, placebo-controlled randomised trial of octreotide [abstract]. European Journal of Palliative Care 2017:173; EAPC 2017, 18-20 May, Madrid, Spain.

[16] McCaffrey N, Hardy J, Fazekas B, Agar M, Devilee L, Rowett D, Currow D (2016). Potential economic impact on hospitalisations of the Palliative Care Clinical Studies Collaborative (PaCCSC) ketamine randomised controlled trial. Aust Health Rev.

[17] Cleeland CS, Ryan KM (1994). Pain assessment: global use of the brief pain inventory. Ann Acad Med Singap.

[18] Abernethy AP, Shelby-James T, Fazekas BS, Woods D, Currow DC (2005). The Australia-modified Karnofsky Performance Status (AKPS) scale: a revised scale for contemporary palliative care clinical practice [ISRCTN81117481]. BMC Palliat Care.

[19] Australian New Zealand Clinical Trials Registry (2008). A randomised double blind placebo controlled trial of infusional subcutaneous octreotide in the management of malignant bowel obstruction at the end of life.

[20] Groenvold M, Petersen MA, Aaronson NK, Arraras JI, Blazeby JM, Bottomley A, Fayers PM, de Graeff A, Hammerlid E, Kaasa S, Sprangers MA, Bjorner JB (2006). The development of the EORTC QLQ-C15-PAL: a shortened questionnaire for cancer patients in palliative care. Eur J Cancer.

[21] McCaffrey N, Skuza P, Breaden K, Eckermann S, Hardy J, Oaten S, Briffa M, Currow D (2014). Preliminary development and validation of a new end-of-life patient-reported outcome measure assessing the ability of patients to finalise their Affairs at the end of life. PLoS One.

[22] Fayers PM, Aaronson NK, Bjordal K, Groenvold M, Curran D, Bottomley A, on behalf of the EORTC Quality of Life Group (2001). The EORTC QLQ-C30 scoring manual.

[23] Groenvold M, Petersen M, on behalf of the EORTC Quality of Life Group (2006). Addendum to the EORTC QLQ-C30 scoring manual: scoring of the EORTC QLQ-C15-PAL.

[24] Moher D, Hopewell S, Schulz KF, Montori V, Gøtzsche PC, Devereaux PJ, Elbourne D, Egger M, Altman DG. CONSORT 2010 explanation and elaboration: updated guidelines for reporting parallel group randomised trials. BMJ. 2010;340. 10.1136/bmj.c869. 10.1136/bmj.c869 PMC2844943 20332511

[25] Schulz KF, Altman DG, Moher D.CONSORT 2010 Statement: updated guidelines for reporting parallel group randomised trials. BMJ. 2010;340:c332. https://www.bmj.com/content/340/bmj.c332. 10.1136/bmj.c332 PMC2844940 20332509

[26] Biering K, Hjollund NH, Frydenberg M (2015). Using multiple imputation to deal with missing data and attrition in longitudinal studies with repeated measures of patient-reported outcomes. Clin Epidemiol.

[27] White IR, Royston P, Wood AM (2011). Multiple imputation using chained equations: issues and guidance for practice. Stat Med.

[28] King MT (1996). The interpretation of scores from the EORTC quality of life questionnaire QLQ-C30. Qual Life Res.

[29] Osoba D, Rodrigues G, Myles J, Zee B, Pater J (1998). Interpreting the significance of changes in health-related quality-of-life scores. J Clin Oncol.

[30] Raman S, Ding K, Chow E, Meyer RM, Nabid A, Chabot P, Coulombe G, Ahmed S, Kuk J, Dar AR, Mahmud A, Fairchild A, Wilson CF, Wu JSY, Dennis K, DeAngelis C, Wong RKS, Zhu L, Brundage M (2016). Minimal clinically important differences in the EORTC QLQ-BM22 and EORTC QLQ-C15-PAL modules in patients with bone metastases undergoing palliative radiotherapy. Qual Life Res.

[31] Steinmann D, Paelecke-Habermann Y, Geinitz H, Aschoff R, Bayerl A, Bolling T, Bosch E, Bruns F, Eichenseder-Seiss U, Gerstein J, Gharbi N, Hagg J, Hipp M, Kleff I, Muller A, Schafer C, Schleicher U, Sehlen S, Theodorou M, Wypior H-J, Zehentmayr F, van Oorschot B, Vordermark D (2012). Prospective evaluation of quality of life effects in patients undergoing palliative radiotherapy for brain metastases. BMC Cancer.

[32] Pallant J (2011). SPSS survival manual: a step by step guide to data analysis using SPSS.

[33] Bell KE, Snijders T, Zulyniak M, Kumbhare D, Parise G, Chabowski A, Phillips SM (2017). A whey protein-based multi-ingredient nutritional supplement stimulates gains in lean body mass and strength in healthy older men: a randomized controlled trial. PLoS One.

[34] Stroup W (2013). Inference part II: covariance components. Generalized linear mixed models: modern concepts, methods and applications.

[35] McCaffrey N, Kaambwa B, Currow DC, Ratcliffe J (2016). Health-related quality of life measured using the EQ-5D-5L: south Australian population norms. Health Qual Life Outcomes.

[36] McCaffrey N, Bradley S, Ratcliffe J, Currow DC (2016). What aspects of quality of life are important from palliative care patients’ perspectives? A systematic review of qualitative research. J Pain Symptom Manage.

[37] Lien K, Zeng L, Nguyen J, Cramarossa G, Culleton S, Caissie A, Lutz S, Chow E (2011). Comparison of the EORTC QLQ-C15-PAL and the FACIT-Pal for assessment of quality of life in patients with advanced cancer. Expert Rev Pharmacoecon Outcomes Res.

