# Supplementary material for: Health-related quality of life in patients with inoperable malignant bowel obstruction: secondary outcome from a double-blind, parallel, placebo-controlled randomised trial of octreotide
Source: BMC Cancer. 2020 Oct 31;20:1050. doi: 10.1186/s12885-020-07549-y (PMC7603764; doi:10.1186/s12885-020-07549-y)
Supplement: Supplementary file 1 — Additional file 1: Table A1. Baseline and post-treatment EORTC QLQ-C15-PAL mean scores (multiple imputation). Baseline and post-treatment EORTC QLQ-C15-PAL mean scores for completed items and multiple imputation. [file 12885_2020_7549_MOESM1_ESM.docx]

**Table A1 Baseline and post-treatment EORTC QLQ-C15-PAL mean scores (multiple imputation)**

|  | **Octreotide** | **Octreotide N=52 (MI)** | **Octreotide** | **Octreotide N=52 (MI)** | **Placebo** | **Placebo N=54 (MI)** | **Placebo** | **Placebo N=54 (MI)** |
| --- | --- | --- | --- | --- | --- | --- | --- | --- |
| **EORTC QLQ-C15-PAL scale*** | **Baseline**  **Mean** | | **Post-treatment**  **Mean** | | **Baseline**  **Mean** | | **Post-treatment**  **Mean** | |
| Overall quality of life^#^ | 22.08  n=40 | 23.32 | 30.81  n=33 | 33.80 | 31.48  n=36 | 31.59 | 32.22  n=30 | 34.27 |
| Physical functioning^#^ | 27.69  n=39 | 26.96 | 32.44  n=30 | 32.39 | 31.17  n=40 | 30.31 | 30.00  n=30 | 31.48 |
| Emotional functioning^#^ | 57.65  n=36 | 59.02 | 71.80  n=26 | 66.39 | 55.25  n=35 | 56.41 | 63.22  n=29 | 63.33 |
| Fatigue^^^ | 74.09  n=39 | 73.26 | 71.88  n=32 | 69.45 | 74.10  n=39 | 72.40 | 71.88  n=32 | 65.76 |
| Nausea & vomiting^^^ | 73.18  n=41 | 72.57 | 40.70  n=34 | 39.50 | 60.53  n=38 | 61.41 | 35.95  n=32 | 38.42 |
| Pain^^^ | 60.16  n=41 | 60.72 | 43.94  n=33 | 46.23 | 55.42  n=40 | 56.00 | 42.93  n=33 | 45.46 |
| Dyspnoea^^^ | 40.65  n=41 | 40.93 | 32.32  n=33 | 34.20 | 36.75  n=39 | 37.41 | 27.08  n=32 | 31.51 |
| Insomnia^^^ | 45.83  n=40 | 45.51 | 33.33  n=33 | 35.77 | 46.67  n=40 | 46.73 | 47.92  n=32 | 46.27 |
| Appetite loss^^^ | 74.17  n=40 | 72.79 | 56.25  n=32 | 53.85 | 82.05  n=39 | 80.80 | 57.58  n=33 | 56.98 |
| Constipation^^^ | 67.50  n=40 | 66.12 | 50.00  n=34 | 48.88 | 67.50  n=40 | 66.51 | 52.53  n=33 | 53.25 |

Table notes: HrQoL = health-related quality of life; MI = multiple imputation; ^#^ higher scores represent better outcomes; ^^^ higher scores represent greater symptom burden
