# Supplementary material for: Health-related quality of life in patients with inoperable malignant bowel obstruction: secondary outcome from a double-blind, parallel, placebo-controlled randomised trial of octreotide
Source: BMC Cancer. 2020 Oct 31;20:1050. doi: 10.1186/s12885-020-07549-y (PMC7603764; doi:10.1186/s12885-020-07549-y)
Supplement: Supplementary file 2 — Additional file 2: Table A2. Baseline and post-treatment EORTC QLQ-C15-PAL completion rates. [file 12885_2020_7549_MOESM2_ESM.docx]

**Table A2: Proportion of respondents with EORTC QLQ-C15-Pal QOL, nausea, pain and total scores and individual item response rates**

|  | **Baseline** | | **Post-treatment** | | **Both** | |
| --- | --- | --- | --- | --- | --- | --- |
|  | **Octreotide (N=52)** | **Placebo**  **(N=54)** | **Octreotide (N=52)** | **Placebo (N=54** | **Octreotide (N=52)** | **Placebo (N=54)** |
| **Score** | | | | | | |
| HrQOL (%) | 40 (76.9) | 36 (66.7) | 33 (63.5) | 30 (55.6) | 32 (61.5) | 23 (42.6) |
| Nausea (%) | 41 (78.8) | 38 (70.4) | 34 (65.4) | 32 (59.3) | 33 (63.5) | 26 (48.1) |
| Pain (%) | 41 (78.8) | 40 (74.1) | 33 (63.5) | 33 (61.1) | 32 (61.5) | 27 (50.0) |
| **Items** | | | | | | |
| All items | 35 (67.3) | 30 (55.6) | 24 (46.2) | 28 (51.9) | 23 (44.2) | 19 (35.2) |
| 1 | 39 (75.0) | 39 (72.2) | 30 (57.7) | 31 (57.4) | 29 (55.8) | 24 (44.4) |
| 2 | 40 (76.9) | 39 (72.2) | 31 (59.6) | 31 (57.4) | 30 (57.7) | 24 (44.4) |
| 3 | 40 (76.9) | 39 (72.2) | 32 (61.5) | 30 (55.6) | 31 (59.6) | 23 (42.6) |
| 4 | 41 (78.8) | 38 (70.4) | 33 (63.5) | 32 (59.3) | 32 (61.5) | 25 (46.3) |
| 5 | 41 (78.8) | 39 (72.2) | 33 (63.5) | 32 (59.3) | 32 (61.5) | 25 (46.3) |
| 6 | 40 (76.9) | 39 (72.2) | 33 (63.5) | 32 (59.3) | 31 (59.6) | 25 (46.3) |
| 7 | 41 (78.8) | 39 (72.2) | 32 (61.5) | 32 (59.3) | 31 (59.6) | 25 (46.3) |
| 8 | 40 (76.9) | 38 (70.4) | 32 (61.5) | 33 (61.1) | 30 (57.7) | 25 (46.3) |
| 9 | 41 (78.8) | 37 (68.5) | 34 (65.4) | 32 (59.3) | 33 (63.5) | 25 (46.3) |
| 10 | 40 (76.9) | 39 (72.2) | 34 (65.4) | 33 (61.1) | 33 (63.5) | 26 (48.1) |
| 11 | 39 (75.0) | 38 (70.4) | 34 (65.4) | 32 (59.3) | 32 (61.5) | 24 (44.4) |
| 12 | 40 (76.9) | 38 (70.4) | 31 (59.6) | 30 (55.6) | 30 (57.7) | 23 (42.6) |
| 13 | 36 (69.2) | 35 (64.8) | 27 (51.9) | 29 (53.7) | 26 (50.0) | 21 (38.9) |
| 14 | 37 (71.2) | 36 (66.7) | 31 (59.6) | 30 (55.6) | 30 (57.7) | 21 (38.9) |
| 15 | 40 (76.9) | 35 (64.8) | 33 (63.5) | 30 (55.6) | 32 (61.5) | 22 (40.7) |

HrQOL = health-related quality of life
